# Long non-coding RNA AFAP1-AS1 accelerates the progression of melanoma by targeting miR-653-5p/RAI14 axis

**DOI:** 10.1186/s12885-020-6665-2

**Published:** 2020-03-30

**Authors:** Fei Liu, Lanting Hu, Yi Pei, Ke Zheng, Wei Wang, Shenglong Li, Enduo Qiu, Guanning Shang, Jiaming Zhang, Xiaojing Zhang

**Affiliations:** 1 Department of Bone and Soft Tissue Tumor Surgery, Cancer Hospital of China Medical University, Liaoning Cancer Hospital & Institute, No.44 Xiaoheyan Road, Dadong District, Shenyang, 110000 Liaoning China; 2 Department of Dermatology, the Seventh People’s Hospital of Shenyang, Shenyang, 110000 Liaoning Province China

**Keywords:** AFAP1-AS1, miR-653-5p, RAI14, Melanoma

## Abstract

**Background:**

Melanoma is the most aggressive skin cancer that derived from pigment cells, accounting for the majority of the skin-cancer-related deaths. Despite great development and evolution have been made in surgery, radiotherapy and adjuvant chemotherapy, the prognosis of melanoma patients exhibited no significant improvement. Long noncoding RNAs (lncRNAs) are frequently dysregulated and involved in the development of cancers. LncRNA AFAP1-AS1 has been explored in various cancers, whereas its role and regulatory mechanism in melanoma are not well understood.

**Methods:**

The expression of AFAP1-AS1 was detected by qRT-PCR. CCK-8, colony formation, transwell and western blot assays were performed to investigate the biological role of AFAP1-AS1 in melanoma. Male BALB/c nude mice were applied for in vivo experiments. The interaction among AFAP1-AS1, miR-653-5p and RAI14 was investigated by RNA pull down, RIP and luciferase reporter assays.

**Results:**

AFAP1-AS1 was highly expressed in melanoma cell lines. Suppression of AFAP1-AS1 impaired cell proliferation, migration, invasion and EMT in melanoma. Moreover, AFAP1-AS1 was a ceRNA of RAI14 by competitively binding with miR-653-5p. Besides, miR-653-5p overexpression or RAI14 inhibition could repress tumor growth. Eventually, rescue assays indicated that the function of AFAP1-AS1 in the cellular process of melanoma was dependent on miR-653-5p and RAI14.

**Conclusions:**

AFAP1-AS1 exerts its oncogenic function in melanoma by targeting miR-653-5p/RAI14 axis.

## Background

Melanoma is the most aggressive skin cancer that derived from pigment cells, accounting for the majority of skin-cancer-related deaths [1, 2]. Melanoma is featured in rapid progression and metastasis [3]. Despite the great development and evolution in surgery, radiotherapy and adjuvant chemotherapy, the prognosis of melanoma patients is still disappointing [4–6]. Therefore, it is necessary to find novel treatment strategy for melanoma. Elucidating the complicated molecular mechanisms is crucial for the identification of novel biological targets for the application in clinical treatment.

With a length of more than 200 nts, long non-coding RNAs (lncRNAs) are a group of transcripts with very finite potential to encode proteins [7, 8]. Nevertheless, increasing evidences demonstrated that lncRNAs play essential roles in the regulation of cancer biological characteristics, including cell proliferation [9], migration [10], invasion [11] and cell differentiation [12]. The biological involvement of lncRNAs in cancers has been investigated in many documents [13]. LncRNA AFAP1 antisense RNA 1 (AFAP1-AS1) has been revealed to participate in promoting cancer progression. Up-regulated lncRNA AFAP1-AS1 promotes carcinogenesis of breast cancer and is a molecular biomarker indicating poor prognosis [14]. LncRNA AFAP1-AS1 plays an oncogenic role in promoting cell migration of non-small cell lung cancer [15]. LncRNA AFAP1-AS1 accelerates the proliferation and metastasis of prostate cancer via inhibiting RBM5 expression [16]. Although the carcinogenic role of AFAP1-AS1 has been demonstrated in several cancers [17], the expression and biological role of AFAP1-AS1 in melanoma remain unclear. In recent years, it has been revealed that lncRNAs could act as microRNA (miRNA) sponges, thereby mediating the expression of messenger RNAs (mRNAs) [18]. For instance, LncRNA EPB41L4A-AS2 targets miR-301a-5p/FOXL1 axis to suppress the malignancy of hepatocellular carcinoma [19]. This lncRNA-miRNA-mRNA regulatory axis emerged as the important competing endogenous RNAs (ceRNA) regulatory network at post-transcriptional level. LncRNA LINC00511 sponges miR-185-3p to regulate breast cancer tumorigenesis and stemness [20]. Therefore, we would explore the potential ceRNA regulatory network involving AFAP1-AS1 in melanoma.

In the present study, we planned to investigate the function and the potential mechanism of AFAP1-AS1 in melanoma. The impact of AFAP1-AS1 on cellular processes of melanoma cells was estimated via loss-of-function assays. Moreover, the in vivo experiments were utilized to further compensate for exploring tumor growth. Finally, all combined results uncovered that AFAP1-AS1 facilitates the malignancy of melanoma by targeting miR-653-5p/RAI14 axis.

## Methods

### Cell culture

Human epidermal melanocytes HEMa-LP (C0245C) were provided by Thermo Fisher Scientific (Waltham, MA, USA). Four human melanoma cell lines A375 (CRL-1619), M21 (BAA-1539), B16F10 (CRL-6475) and SK-MEL-2 (HTB-68), were obtained from American Type Culture Collection (ATCC; Manassas, VA, USA). All cell lines were cultured at 37 °C with 5% CO_2_. Then, cells were all maintained continuously in DMEM medium (Thermo Fisher Scientific) supplied with 10% fetal bovine serum (FBS; Thermo Fisher Scientific) and 1% antibiotics (Invitrogen, Carlsbad, CA, USA).

### RNA isolation and qRT-PCR

Using the TRIzol Reagent (Invitrogen), total RNAs were extracted from cells, which were reverse transcribed to generate first-stand cDNA. Next, qRT-PCR was carried out using SYBR® Premix Ex Taq™ II kit (Takara, Dalian, China) on 7500 Real-Time PCR System (Applied Biosystems, Foster City, CA, USA) to determine the quantification of AFAP1-AS1 expression. Results were calculated by the 2^-ΔΔCt^ method, followed by normalization to GAPDH/U6.

### Cell transfection

Under the standard conditions, A375 or M21 cells were incubated and seeded into 6-well plates. The lentivirus vector bearing short hairpin RNAs (shRNAs) sequences targeting AFAP1-AS1 (sh-AFAP1-AS1#1/2), RAI14 (sh-RAI14#1/2), their corresponding negative control (sh-NC), miR-653-5p mimics/inhibitors, NC mimics/inhibitors, the RAI14 overexpressing plasmids and empty pcDNA3.1 vector were constructed by GenePharma (Shanghai, China). Using Lipofectamine 2000 (Invitrogen), transfection of above plasmids was implemented in A375 or M21 cells. 48 h lately, transfected cells were collected.

### CCK-8 assay

Transfected A375 or M21 cells were seeded in 96-well plates and then incubated. Subsequently, the same amount of CCK-8 solution was added to each well. Cells were then incubated for another 4 h. The absorbance values were tested by Thermo-max microplate reader (Thermo Fisher Scientific) at 450 nm.

### Colony formation assay

After being placed into 6-well plates, transfected A375 or M21 cells were incubated for 14 days. Following that, cells were fixed by paraformaldehyde (PFA; Sigma-Aldrich, St. Louis, MO, USA) and stained by crystal violet solution (Sigma-Aldrich). Colonies in each well were visualized, followed by quantitated via Image J software (Brainlab, Munich, Germany).

### Transwell assay

In order to determine cell migration, a 24-well chamber containing an aperture of 8 μm was employed. Transfected A375 or M21 cells were inoculated into the top chamber filled with 300 μl serum-free medium. DMEM (Thermo Fisher Scientific) with 10% FBS was added into the lower chamber. After incubating 1 day, remained cells from the upper surface were treated with a cotton swab in the top chamber. Cells that migrated to the bottom of the membrane were washed and fixed with 4% paraformaldehyde, then dyed. Finally, migrated cells were counted via a 200 × microscope (Olympus, Tokyo, Japan).

For cell invasion, the condition of culture is no difference. However, the membrane of the chamber was coated with Matrigel solution (BD Diagnostics, Franklin Lakes, NJ). 24 h later, cells that invaded to the lower chambers were pictured and counted under a microscope.

### Western blot

Total proteins were isolated from A375 or M21 cells and the concentration was detected via BCA Protein Assay Kit (Takara, Tokyo, Japan). Protein samples in equal quantity were separated via sodium dodecyl sulfate-polyacrylamide gel electrophoresis (SDS-PAGE) (Millipore, Bedford, MA, USA), and then transferred into the PVDF (Millipore) membranes. Membranes blocked with non-fat milk were incubated with primary antibodies from Abcam (Cambridge, USA) of anti-E-cadherin (ab194982), anti-N-cadherin (ab202030), anti-RAI14 (ab137118), anti-Ki67 (ab16667) and anti-GAPDH (ab8245), followed by with secondary antibody. GAPDH was regarded to be an endogenous control. Western bands were observed using ECL detection system.

### Tumor Xenograft model

Thirty-six male BALB/c nude mice (age: 5 weeks ±1 week; weight: 23 g ± 2 g) obtained from the National Laboratory Animal Center (Beijing, China) were fed under the condition of specific pathogen-free. Then, the suspension of A375 cells transfected with sh-NC or sh-AFAP1-AS1#1 (with a concentration of 2 × 10^7^ cells/ml) was subcutaneously injected into lower right flank of each mouse that was randomly divided into two groups (with 6 mice each group). Another group (two subgroups) of 12 mice was injected with cells transfected with NC mimics or miR-653-5p mimics (RiboBio Co., Ltd., Guangzhou, China) in the same way. The left 12 mice were injected with cells transfected with sh-NC or sh-RAI14#1 similarly. Every 4 days, the volumes and weights of tumors were measured and calculated via the formula of length × width^2^ × 0.5. After injection of 4 weeks, mice were euthanized via dislocation of cervical vertebra method and then tumor was excised, followed by extraction for further Ki67 staining. All procedures during the in vivo experiments were approved by the Institutional Committee for Animal Research and kept to the national guidelines for the care and use of laboratory animals (GB14925–2010).

### Fluorescent in situ hybridization (FISH)

Using Ribo™ Fluorescent in Situ Hybridization Kit (RiboBio, Guangzhou, China), FISH assay was performed. Nucleus was stained via DAPI, which were designed and synthesized by Ribobio. Cy3 fluorescent dye was selected to label AFAP1-AS1. Fluorescence detection was conducted via the confocal laser-scanning microscope (Leica Microsystems, Wetzlar, Germany).

### RNA pull-down assay

In short, biotinylated AFAP1-AS1 probe (AFAP1-AS1 probe biotin) or a negative control probe (AFAP1-AS1 probe-no biotin) were separately synthesized from Thermo Fisher Scientific. Next, the biotinylated lncRNA (lncRNA biotin) was transfected into A375 or M21 cells. After 2 days, cells were subjected to RNA pull-down assay. The results were analyzed by qRT-PCR.

### Dual-luciferase reporter assay

The wild-type or mutant binding sequence of miR-653-5p in AFAP1-AS1 or RAI14 3′-UTR was synthesized and sub-cloned into pmirGLO dual-luciferase vector (Promega, Madison, WI, USA). AFAP1-AS1-Wt/Mut vector was co-transfected with miR-653-5p mimics or NC mimics into A375 or M21 cells. Besides, A375 or M21 cells were co-transfected with RAI14-Wt/Mut vector or NC mimics/miR-653-5p mimics/miR-653-5p mimics + pcDNA3.1/AFAP1-AS1. 48 h later, Dual Luciferase Report Assay System (Promega) was employed to monitor luciferase activity.

### RNA Immunopreciptiation (RIP)

After transfection, A375 or M21 cells were cross-linked with formaldehyde (Sigma-Aldrich), and then lysed in RIP lysis buffer (Thermo Fisher Scientific). Subsequently, cells were incubated overnight with magnetic beads (Invitrogen) conjugated to antibodies of anti-Ago2 (Abcam) and anti-IgG (Abcam) at 4 °C. The levels of AFAP1-AS1, miR-653-5p and RAI14 were determined via qRT-PCR.

### Statistical analysis

Results were listed as mean ± standard deviation (SD). All experiments were in triplicate. Statistical analyses were produced using GraphPad Prism 7.0 (GraphPad Software. La Jolla, CA, USA). Unpaired or paired student’s t-test or one-way or two-way ANOVA with post hoc tests (Dunnett or Turkey) was appropriately employed for analysis of differences. *P* < 0.05 had statistically significance.

## Results

### AFAP1-AS1 is highly expressed in melanoma cell lines and enhances melanoma tumorigenesis and development

**Fig. 1:**
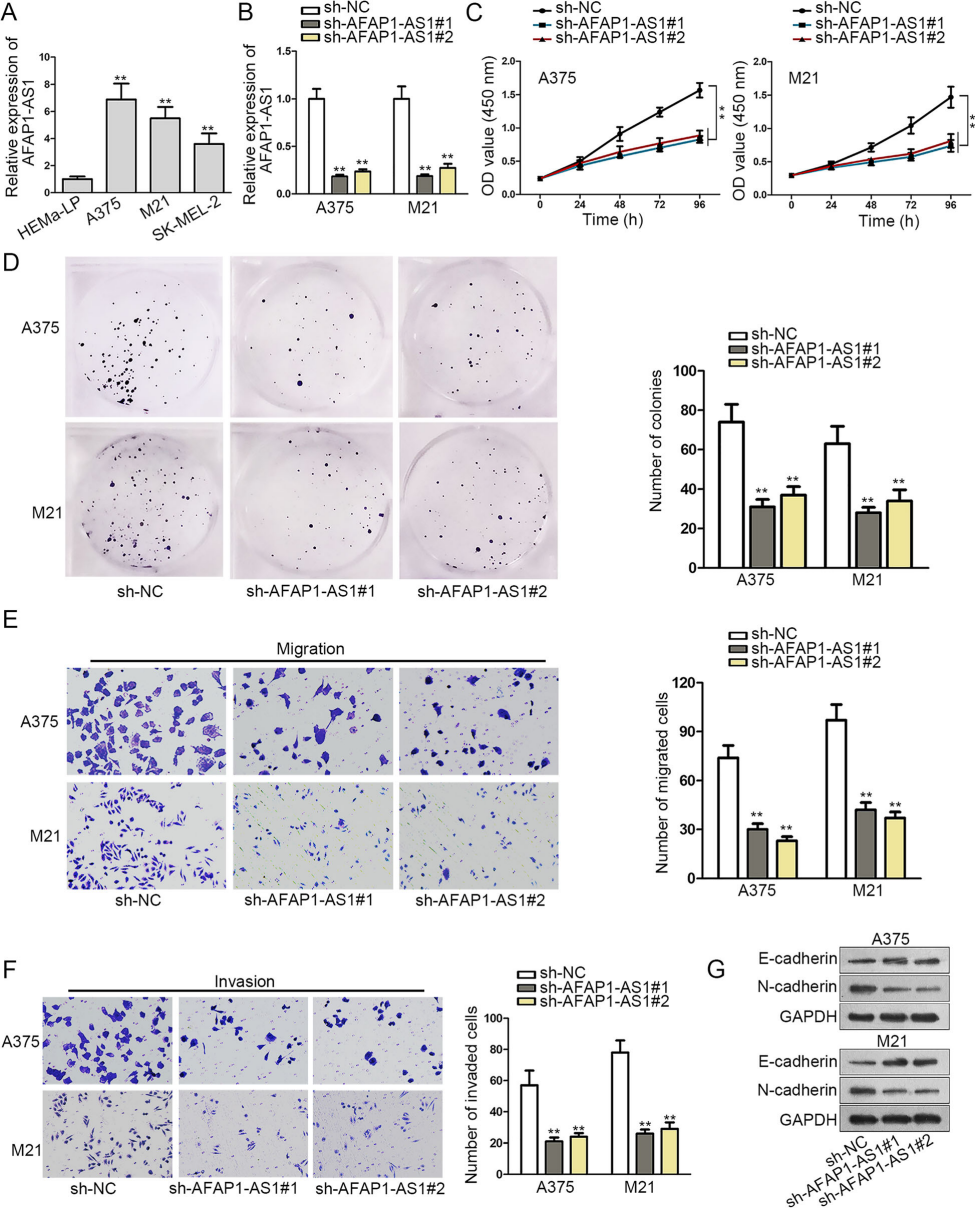
AFAP1-AS1 is highly expressed in melanoma cell lines and enhances melanoma tumorigenesis and development. **a** qRT-PCR assay was conducted to detect the expression of AFAP1-AS1 in melanoma cells and in normal human epidermal melanocyte HEMa-LP cells. **b** qRT-PCR assay evaluated the knockdown efficiency of sh-AFAP1-AS1#1/2. **c**-**d** CCK-8 and colony formation assay reflected cell proliferation. **e-f** Transwell assays studied cell migration and invasion. **g** Western blot assay was applied to measure the expression of EMT process related proteins (E-cadherin and N-cadherin) in A375 and M21 cells. ^**^P < 0.01

Firstly, we studied the potential role of AFAP1-AS1 in melanoma. qRT-PCR results showed that AFAP1-AS1 exhibited conspicuous high expression in melanoma cell lines (A375, M21, B16F10 and SK-MEL-2) in comparison to normal human epidermal melanocyte HEMa-LP cells (Fig. 1a). The expression of AFAP1-AS1 in A375 and M21 cells remarkably declined by the transfection of sh-AFAP1-AS1#1/2 (Fig. 1b). Through CCK-8 and colony formation assay, we discovered that the cell proliferation was inhibited by suppressing AFAP1-AS1 (Fig. 1c-d). Transwell assay was conducted to detect the migrating and invasive abilities of melanoma cells. We found that the migrating and invasive abilities of A375 and M21 cells were impaired by AFAP1-AS1 knockdown (Fig. 1e-f). Western blot assay showed that depletion of AFAP1-AS1 notably enhanced E-cadherin expression and reduced N-cadherin expression (Fig. 1g). To further explore the role of AFAP1-AS1 in melanoma, in vivo assays were performed using healthy nude mice (weight: 23 g ± 2 g; no infection and any treatment previously). The tumor size, volume and weight (analyzed using 6 animals in each group) were measured and found down-regulated in mice inoculated with sh-AFAP1-AS1#1 (Fig. S1A-B). AFAP1-AS1 was significantly lowly expressed in mice inoculated with sh-AFAP1-AS1#1 (Fig. S1C). In addition, E-cadherin expression was elevated, while N-cadherin and Ki67 expression was decreased in response to AFAP1-AS1 repression (Fig. S1D). These results demonstrated that AFAP1-AS1 is up-regulated in melanoma cell lines and facilitates melanoma cells growth, migration, invasion and EMT process.

### AFAP1-AS1 directly binds to miR-653-5p and represses miR-653-5p expression

**Fig. 2:**
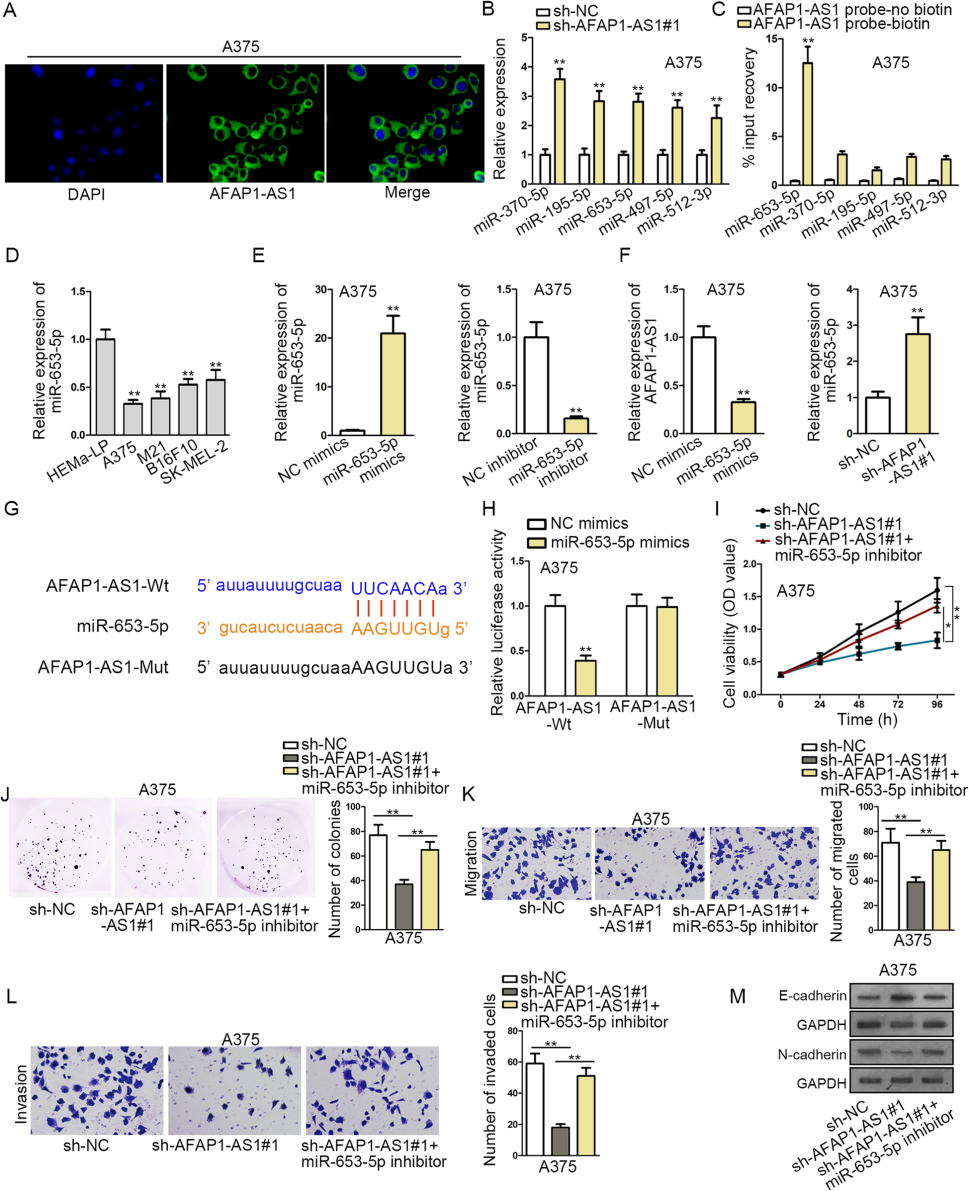
AFAP1-AS1 directly binds to miR-653-5p and represses miR-653-5p expression. **a** FISH assay was used to confirm the location of AFAP1-AS1 in A375 cells. **b** 29 miRNAs were screened out by ENCORI (The Encyclopedia of RNA Interactomes) website to have binding sites for AFAP1-AS1. qRT-PCR assay detected the up-regulation of miRNAs (miR-653-5p, miR-370-5p, miR-195-5p, miR-497-5p and miR-512-3p) by AFAP1-AS1 knockdown. **c** The binding ability of miRNAs to AFAP1-AS1 was verified through RNA pull down assay. **d** The expression of miR-653-5p in melanoma cells was measured by qRT-PCR assay. **e** qRT-PCR assay explored the overexpression and knockdown efficiency of miR-653-5p. **f** qRT-PCR assay investigated the relationship between the expression level of AFAP1-AS1 and miR-653-5p. **g** There were the binding sites between AFAP1-AS1 and miR-653-5p. **h** Luciferase reporter assay was conducted to verify the binding ability between AFAP1-AS1 and miR-653-5p. **i-j** CCK-8 and colony formation assay disclosed cell proliferation. **k-l** Cell migration and invasion were observed in transwell assays. **m** Western blot assay was applied to measure the expression of EMT process related proteins. ^*^P < 0.05, ^**^P < 0.01

LncRNAs have been reported to modulate gene expression in multifold mechanisms. Next, we further investigated the regulatory mechanism related to AFAP1-AS1. We firstly studied the subcellular distribution of AFAP1-AS1 in A375 and M21 cells. FISH assay revealed that AFAP1-AS1 was mainly located in the cytoplasm of A375 and M21 cells (Fig. 2a, Fig. S1E). We speculated that AFAP1-AS1 may realize its biological function by acting as a ceRNA. Through ENCORI (The Encyclopedia of RNA Interactomes) website, we found 29 miRNAs have binding sites for AFAP1-AS1. qRT-PCR assay was performed to detect the expression of miRNAs in sh-NC or sh-AFAP1-AS1#1 groups, and 5 miRNAs that up-regulated in sh-AFAP1-AS1#1 transfected cells were shown in Fig. 2b and Fig. S1F. RNA pull down assay was used to confirm the binding ability between AFAP1-AS1 and miRNAs. Results showed that only miR-653-5p was significantly enriched by biotinylated AFAP1-AS1 probe (Fig. 2c, Fig. S1G). In subsequence, we observed that miR-653-5p was down-regulated in melanoma cells (Fig 2d). Then, the overexpression and knockdown efficiency of miR-653-5p was detected by qRT-PCR (Fig. 2e, Fig. S1H). MiR-653-5p overexpression decreased AFAP1-AS1 expression, whereas AFAP1-AS1 repression increased miR-653-5p expression (Fig. 2f, Fig. S1I). We found the putative binding sites between AFAP1-AS1 and miR-653-5p, as demonstrated in Fig. 2g. Luciferase reporter assay has been utilized in the confirmation of the affinity between genes [19, 20]. If gene A could interact with gene B, the luciferase activity of the pmirGLO vector containing the wild type binding sites of gene A on gene B could be weakened by the overexpression of gene A. As depicted in Fig. 2h and Fig. S1J, the luciferase activity of AFAP1-AS1-Wt was impaired by miR-653-5p mimics, while that of AFAP1-AS1-Mut was unaffected by miR-653-5p mimics, verifying the combination between AFAP1-AS1 and miR-653-5p. Rescue assays revealed that the decrease of cell proliferation induced by AFAP1-AS1 deficiency could be reversed by miR-653-5p silencing (Fig. 2i-j). The migrating and invasive abilities were blocked by repressing AFAP1-AS1, but this effect was countervailed by miR-653-5p inhibition (Fig. 2k-l). MiR-653-5p knockdown could rescue the increase of E-cadherin expression and the decrease of N-cadherin expression caused by AFAP1-AS1 knockdown (Fig. 2m). As shown in Fig. S2A-C, the tumor size, volume and weight were repressed by miR-653-5p overexpression. Moreover, the expression of miR-653-5p was elevated in mice transfected with miR-653-5p mimics (Fig. S2D). In the end, western blot assay indicated that E-cadherin expression was increased when miR-653-5p was up-regulated, and N-cadherin as well as Ki67 levels were inhibited by miR-653-5p overexpression (Fig. S2E). Collectively, AFAP1-AS1 directly binds to miR-653-5p and miR-653-5p overexpression suppresses melanoma progression in vivo.

### AFAP1-AS1 absorbs miR-653-5p to regulate RAI14

**Fig. 3:**
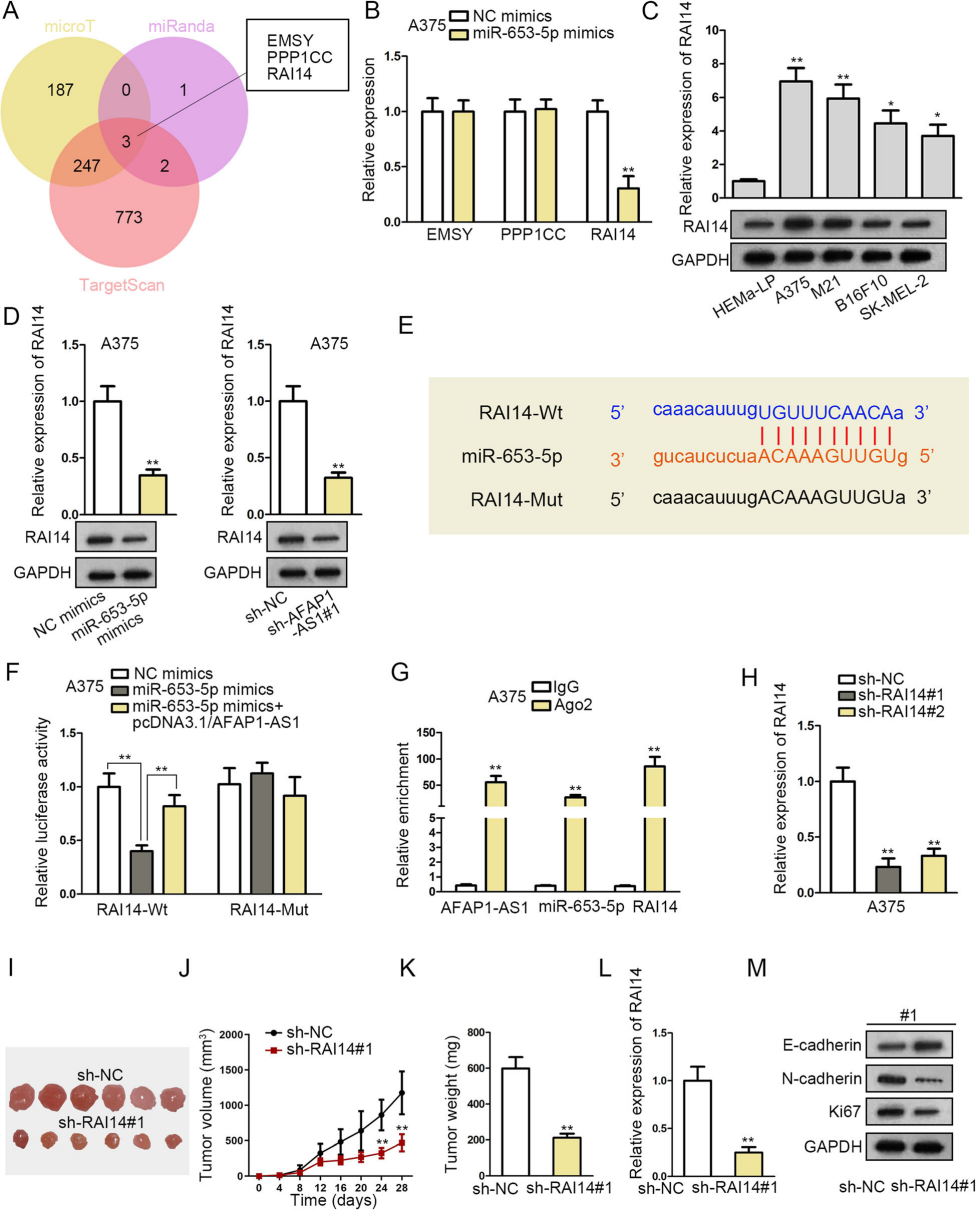
AFAP1-AS1 absorbs miR-653-5p to regulate RAI14. **a** Venn diagram (data predicted by microT, miRanda and TargetScan bioinformatics tools) showed 3 probable genes that may interact with miR-653-5p. **b** qRT-PCR assay examined the expression of 3 mRNAs in miR-653-5p mimics-transfected cells. **c** qRT-PCR and western blot assays detected the expression of RAI14 in melanoma cells. **d** qRT-PCR and western blot assays unveiled the expression of RAI14 after overexpressing miR-653-5p or suppressing AFAP1-AS1. **e** The binding sites between RAI14 and miR-653-5p. **f** Luciferase reporter assay assessed the binding ability among AFAP1-AS1, miR-653-5p and RAI14. **g** RIP assay studied the interaction among AFAP1-AS1, miR-653-5p and RAI14. **h** The interference efficiency of RAI14 was evaluated by qRT-PCR. **i** The images of the tumors injected with sh-NC or sh-RAI14#1. **j** The volume of tumors was recorded. **k** The weight of tumors was measured. **l** qRT-PCR detected RAI14 mRNA level in the tumor xenografts. **m** Western blot assay examined Ki67, E-cadherin and N-cadherin protein expressions in sh-NC group or sh-RAI14#1 group in A375 cells collected from tumors. ^*^P < 0.05, ^**^P < 0.01

Through microT, miRanda and TargetScan bioinformatics tools, we screened out 3 probable genes that may interact with miR-653-5p, as displayed in the Venn diagram (Fig. 3a). Overexpression of miR-653-5p could lead to evident decline on the expression of RAI14 (Fig. 3b, Fig. S2F). Besides, RAI14 was revealed to be markedly high-expressed in melanoma cells (Fig. 3c). MiR-653-5p overexpression or AFAP1-AS1 repression reduced the mRNA and protein expressions of RAI14 (Fig. 3d, Fig. S2G). The corresponding sequences between miR-653-5p and RAI14 were exhibited in Fig. 3e. Result of luciferase reporter assay exhibited that miR-653-5p up-regulation-induced reduction on the luciferase activity of RAI14-Wt could be rescued by overexpressing AFAP1-AS1 (Fig. 3f, Fig. S2H). Finally, RIP assay was performed to study the molecular interplay among AFAP1-AS1, miR-653-5p and RAI14. Ago2 is an essential component of RNA-induced silencing complex (RISC) and therefore RISC could be pulled by antibody targeting Ago2. Besides, mRNAs and lncRNAs that could bind with miRNA all existed in RISC. Results demonstrated that AFAP1-AS1, miR-653-5p and RAI14 were enriched by antibody targeting Ago2 indicating their coexistence in the miR-653-5p induced silencing complex (Fig. 3g, Fig. S2I). In addition, the expressions of AFAP1-AS1 and miR-653-5p exhibited no change in response to RAI14 overexpression in A375 cells (Fig. S2J-K). Afterwards, to study the role of RAI14 in melanoma, RAI14 was effectively knocked down by sh-RAI14#1/2 for conducting loss-of-function assays (Fig. 3h). We noticed that RAI14 depletion remarkably restrained the tumor size, volume and weight of mice (Fig. 3i-k). Furthermore, the mRNA level of RAI14, the protein level of N-cadherin and Ki67 were detected to be down-regulated by RAI14 silencing in mice. However, the protein expression of E-cadherin was promoted via RAI14 deficiency (Fig. 3l-m, Fig. S3A). All in all, these findings unveiled that RAI14 binds with miR-653-5p and its depletion impairs tumor growth.

### AFAP1-AS1/miR-653-5p/RAI14 axis is involved in melanoma progression

**Fig. 4:**
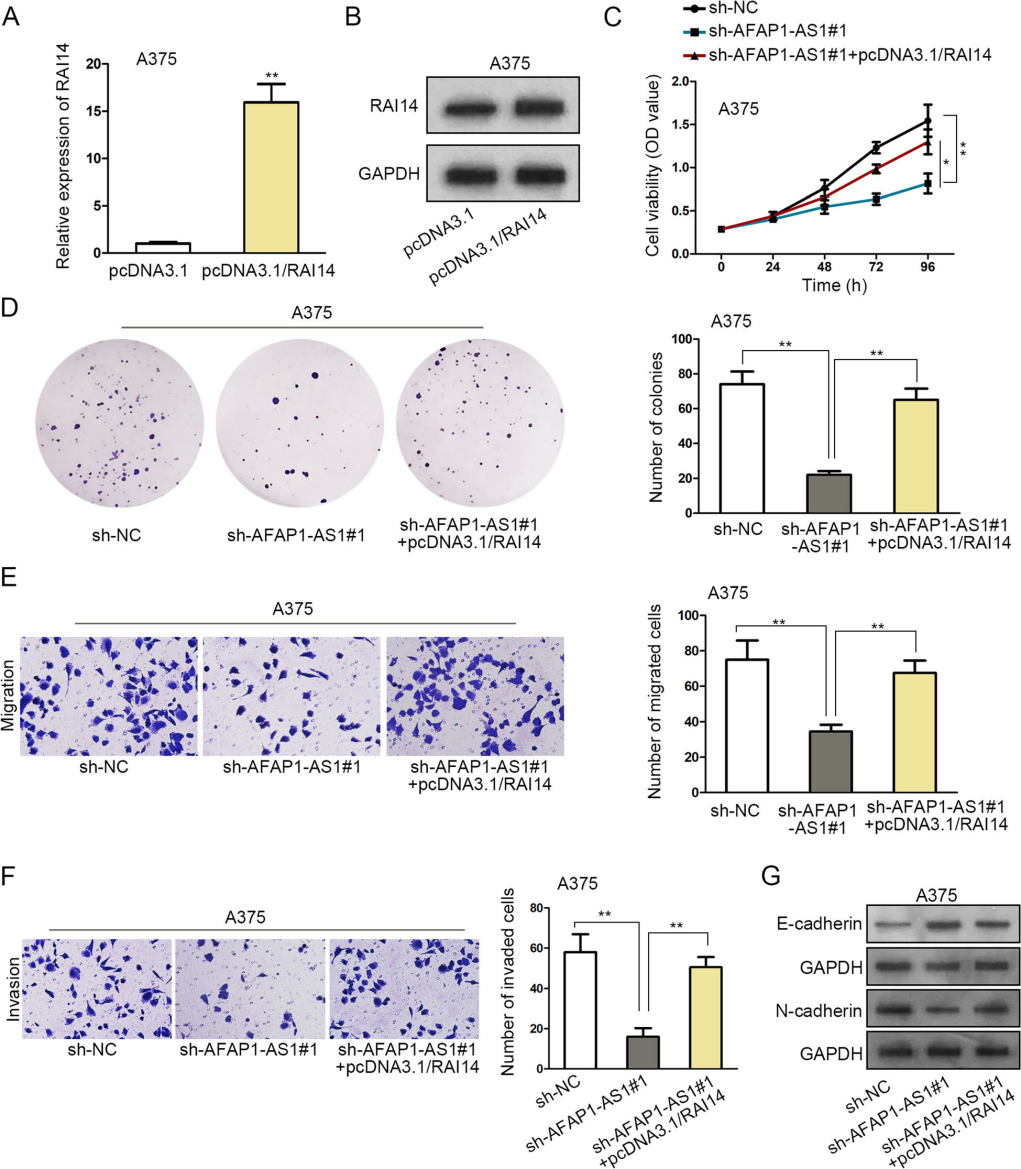
AFAP1-AS1/miR-653-5p/RAI14 axis is involved in melanoma progression. **a** qRT-PCR analyzed RAI14 mRNA expression in pcDNA3.1 or pcDNA3.1/RAI14-transfected cells. **b** Western blot assay revealed the expression of RAI14 in pcDNA3.1 or pcDNA3.1/RAI14-transfected cells. **c** CCK-8 assay reflected cell proliferation at indicated times in transfected cells. **d** Colony formation assay was performed to disclose colongenic ability of transfected cells. **e** Cell migration in different groups was observed in transwell assay. **f** Transwell assay explored cell invasion. **g** Western blot assay was utilized to measure the expression of EMT process related proteins. ^*^P < 0.05, ^**^P < 0.01

To confirm whether AFAP1-AS1/miR-653-5p/RAI14 axis was involved in melanoma progression, rescue assays were conducted. The mRNA and protein expressions of RAI14 were dramatically increased by RAI14 up-regulation (Fig. 4a-b). The cell proliferation was weakened by knocking down AFAP1-AS1, but this effect was counteracted by RAI14 overexpression (Fig. 4c-d). Cell migration and invasion phenotypes were weakened by AFAP1-AS1 depletion, while strengthened in response to RAI14 overexpression (Fig. 4e-f). Up-regulation of RAI14 could abolish the interfering impacts of AFAP1-AS1 depletion on EMT progress, as evidenced by the altered expressions of E-cadherin and N-cadherin (Fig. 4 g). Therefore, it was safe to reach the conclusion that AFAP1-AS1 elicited cellular function changes of melanoma cells via targeting the miR-653-5p/RAI14 axis.

## Discussion

It has been revealed that lncRNAs are involved in the regulation of various cancers, such as lung cancer [21], osteosarcoma [22], colorectal cancer [23] and breast cancer [24]. LncRNA AFAP1-AS1 has been revealed to play a vital role in promoting cancers progression [14–16]. However, the expression and biological role of AFAP1-AS1 in melanoma still remain poorly studied. In the present work, the upregulated AFAP1-AS1 was detected in melanoma cell lines. Depletion of AFAP1-AS1 could pose suppressive effect on cell proliferation, migration, invasion and EMT process. Meanwhile, AFAP1-AS1 depletion also interfered tumor growth in vivo. The in vivo findings provided potent evidence for AFAP1-AS1 in facilitating tumor growth in melanoma, as the xenografts tumors transfected with sh-AFAP1-AS1 grew slower in mice. Collectively, AFAP1-AS1 acts as a tumor promoter in melanoma progression.

LncRNA regulates gene expression via multifold mechanical involvement. At the transcriptional level, lncRNAs could epigenetically silence mRNAs [25]. While at the post-transcriptional level, apart from cooperating with RNA binding proteins in stabilizing gene expression, lncRNAs could serve as ceRNA and directly bind to miRNAs, therefore modulate the expression of target genes [26–28]. LncRNA PVT1 enhances the viability and invasion of papillary thyroid carcinoma cells by functioning as ceRNA of microRNA-30a to mediate the expression of insulin like growth factor 1 receptor [29]. LncRNA 00152 functions as a ceRNA to regulate NRP1 expression by sponging miRNA-206 in colorectal cancer [30]. Through bioinformatics tools and mechanical experiments, we found that miR-653-5p exhibited strong binding potential to AFAP1-AS1. MiR-653-5p has been reported to participate in cancers progression through engaging in ceRNA network. Circular RNA circ-RAD23B promotes cell growth and invasion by targeting miR-593-3p/CCND2 and miR-653-5p/TIAM1 pathways in non-small cell lung cancer [31]. The SNHG7-miR-653-5p-STAT2 feedback loop has been disclosed in regulating neuroblastoma progression [32]. The physical interaction between AFAP1-AS1 and miR-653-5p was unveiled. Besides, AFAP1-AS1 and miR-653-5p could mutually regulate each other. Inhibition of miR-653-5p abrogated the biological functions of AFAP1-AS1 knockdown on melanoma progression. More importantly, miR-653-5p overexpression could suppress tumor growth in vivo. To conclude, miR-653-5p availability was antagonized for AFAP1-AS1-induced tumorigenesis and development of melanoma.

Furthermore, Retinoic acid-induced protein 14 (RAI14) was predicted as a downstream mRNA of miR-653-5p through bioinformatics website. Intimately associated with NF-κB signaling pathway, RAI14 is a developmentally regulated gene induced by retinoic acid. It has been relatively well-studied to an array of cancers and may be utilized as a promising target for anti-cancer drug invention. RAI14 promotes mTOR-mediated inflammation under inflammatory stress and chemical hypoxia in U87 glioblastoma cell line [33]. Knockdown of RAI14 suppresses the progression of gastric cancer [34]. High expression of RAI14 in gastric cancer is revealed and such expression pattern could be an independent molecular predictor of poor prognosis in gastric cancer patients [35]. In our study, RAI14 was confirmed to bind with miR-653-5p and was down-regulated by miR-186-5p. Subsequently, tumorigenesis in vivo was impaired by RAI14 depletion, as evidence by dampened tumor growth. Eventually, rescue assays indicated that up-regulation of RAI14 observably counteracted the effects of AFAP1-AS1 suppression on melanoma cell proliferation, migration, invasion and EMT process.

The functional role of AFAP1-AS1 in melanoma is in accordance with the findings of its oncogenic properties in other cancers, such as gastric cancer [36], prostate cancer [37] and osteosarcoma [38]. Although the discovery of AFAP1-AS1/miR-653-5p/RAI14 axis may enrich the study of AFAP1-AS1 in cancers, the lack of novelty in mechanistic insight is the major limit of this work, which we would improve in further studies.

## Conclusions

These findings revealed that AFAP1-AS1 aggravates melanoma progression through absorbing miR-653-5p and up-regulating RAI14. The discovery of AFAP1-AS1/miR-653-5p/RAI14 axis may offer new insights into melanoma diagnosis and treatment.

## Supplementary information

**Additional file 1: Supplementary Figure S1.** (A) The picture of the tumors injected with sh-NC or sh-AFAP1-AS1#1. The volume of tumors was recorded. (B) The weight of tumors was detected. (C) The expression of AFAP1-AS1 in the tumor xenografts was examined by qRT-PCR. (D) Western blot assay confirmed Ki67, E-cadherin and N-cadherin protein expression in sh-NC group or sh-AFAP1-AS1#1 group in A375 cells collected from tumors. (E) FISH assay determined the location of AFAP1-AS1 in M21 cells. (F) qRT-PCR assay demonstrated the up-regulation of 5 miRNAs by AFAP1-AS1 depletion. (G) RNA pull down assay studied the binding of miRNAs to AFAP1-AS1. (H) qRT-PCR assay quantified the overexpression and knockdown efficiency of miR-653-5p in M21 cells. (I) qRT-PCR assay investigated the regulation between AFAP1-AS1 and miR-653-5p. (J) Luciferase reporter assay explored the combination between AFAP1-AS1 and miR-653-5p. ^**^P < 0.01.

**Additional file 2: Supplementary Figure S2.** (A) The picture of the tumors injected with NC mimics or miR-653-5p mimics. (B) The volume of tumors in NC mimics or miR-653-5p mimics groups was evaluated. (C) The weight of tumors was examined. (D) qRT-PCR quantified the expression of miR-653-5p in the tumor xenografts. (E) Western blot assay revealed Ki67, E-cadherin and N-cadherin protein expression in NC mimics or miR-653-5p mimics-transfected A375 cells collected from tumors. (F) qRT-PCR assay analyzed 3 mRNAs level in miR-653-5p mimics-transfected cells. (G) qRT-PCR and western blot assays examined the mRNA and protein expression of RAI14 after overexpressing miR-653-5p or suppressing AFAP1-AS1. (H) Luciferase reporter assay researched the affinity among AFAP1-AS1, miR-653-5p and RAI14. (I) RIP assay explored the interaction among AFAP1-AS1, miR-653-5p and RAI14. (J) qRT-PCR detected AFAP1-AS1 expression in pcDNA3.1 or pcDNA3.1/RAI14-transfected cells. (K) qRT-PCR measured miR-653-5p level in pcDNA3.1 or pcDNA3.1/RAI14-transfected cells. ^**^P < 0.01.

**Additional file 3: Supplementary Figure S3.** (A) Western blot assay examined Ki67, E-cadherin and N-cadherin protein expressions in sh-NC group or sh-RAI14#1 group in tumors separated from the other 5 tissues of mice.

**Additional file 4 Supplementary file 1-5** The original western blot data of figure 1G/2M/3C/3D/3 M/4B/4G/S1D/S2E/S2G/S3A were displayed.

## Data Availability

Not applicable.

## Abbreviations

ANOVA: Analysis of variance
BCA: Bicinchoninic acid
ceRNA: Competing endogenous RNAs
DAPI: 4′,6-diamidino-2-phenylindole
DMEM: Dulbecco’s Modified Eagle Medium
ECL: Enhanced chemiluminescence
EMT: Epithelial-mesenchymal transition
GAPDH: Glyceraldehyde-3-phosphate dehydrogenase
miRNA: micro RNA
mRNAs: messenger RNAs
PVDF: Polyvinylidene fluoride
qRT-PCR: quantitative real-time polymerase chain reaction
RAI14: Retinoic acid induced 14
RISC: RNA-induced silencing complex
