# Supplementary figures and images for: Long non-coding RNA AFAP1-AS1 accelerates the progression of melanoma by targeting miR-653-5p/RAI14 axis
Source: BMC Cancer. 2020 Mar 30;20:258. doi: 10.1186/s12885-020-6665-2 (PMC7106910; doi:10.1186/s12885-020-6665-2)

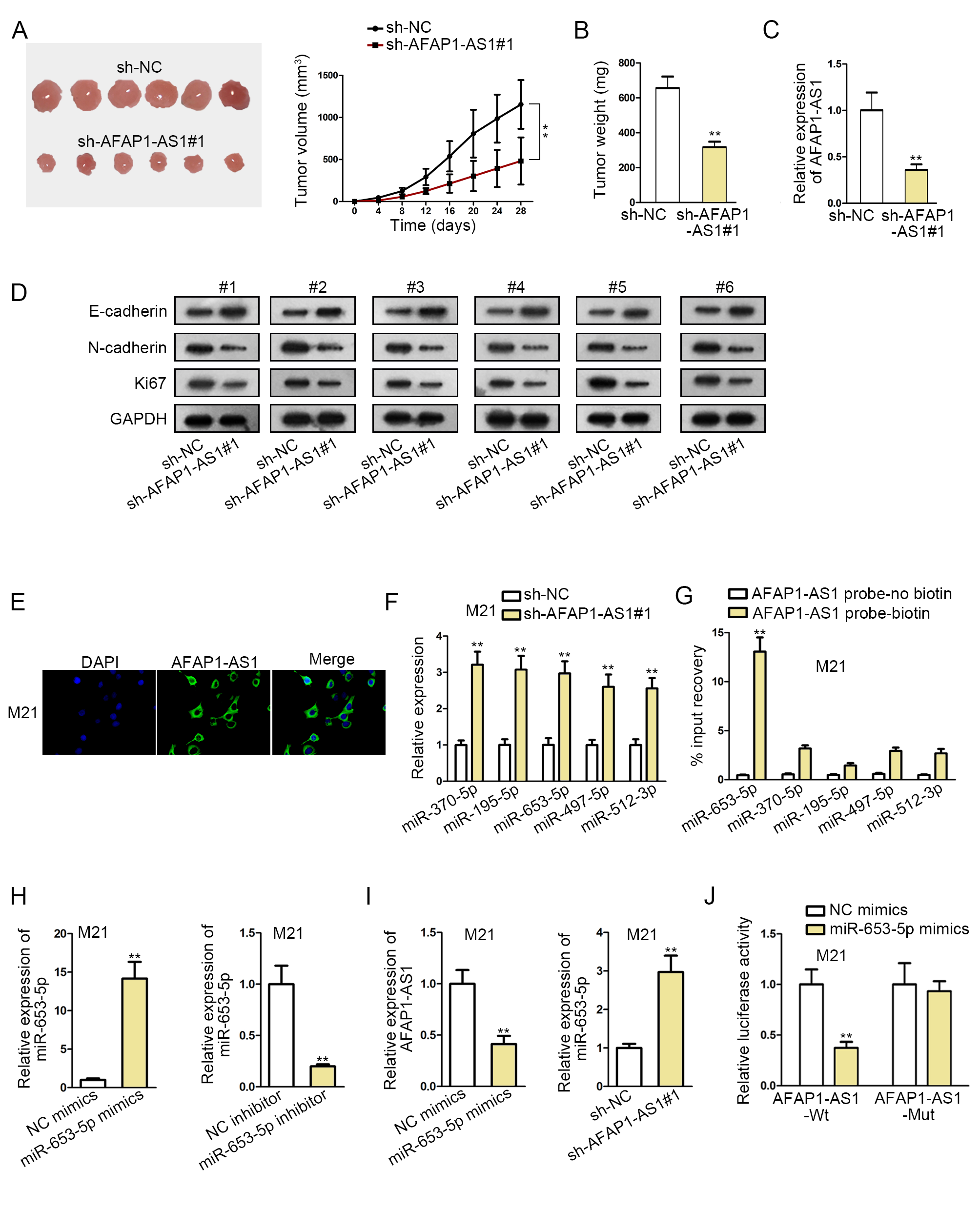

Supplement: Supplementary file 1 — Additional file 1: Supplementary Figure S1. (A) The picture of the tumors injected with sh-NC or sh-AFAP1-AS1#1. The volume of tumors was recorded. (B) The weight of tumors was detected. (C) The expression of AFAP1-AS1 in the tumor xenografts was examined by qRT-PCR. (D) Western blot assay confirmed Ki67, E-cadherin and N-cadherin protein expression in sh-NC group or sh-AFAP1-AS1#1 group in A375 cells collected from tumors. (E) FISH assay determined the location of AFAP1-AS1 in M21 cells. (F) qRT-PCR assay demonstrated the up-regulation of 5 miRNAs by AFAP1-AS1 depletion. (G) RNA pull down assay studied the binding of miRNAs to AFAP1-AS1. (H) qRT-PCR assay quantified the overexpression and knockdown efficiency of miR-653-5p in M21 cells. (I) qRT-PCR assay investigated the regulation between AFAP1-AS1 and miR-653-5p. (J) Luciferase reporter assay explored the combination between AFAP1-AS1 and miR-653-5p. **P < 0.01. [file 12885_2020_6665_MOESM1_ESM.tif]

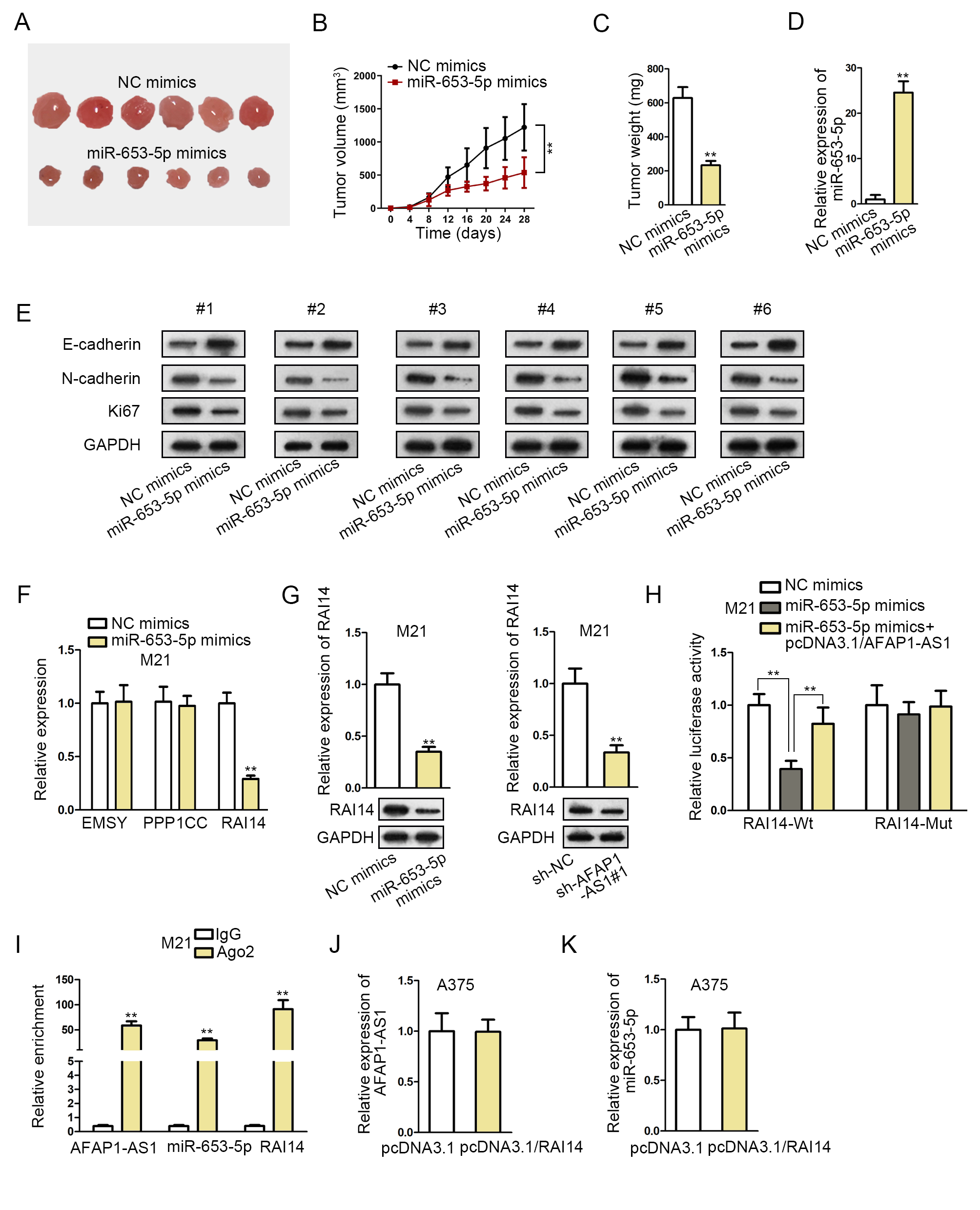

Supplement: Supplementary file 2 — Additional file 2: Supplementary Figure S2. (A) The picture of the tumors injected with NC mimics or miR-653-5p mimics. (B) The volume of tumors in NC mimics or miR-653-5p mimics groups was evaluated. (C) The weight of tumors was examined. (D) qRT-PCR quantified the expression of miR-653-5p in the tumor xenografts. (E) Western blot assay revealed Ki67, E-cadherin and N-cadherin protein expression in NC mimics or miR-653-5p mimics-transfected A375 cells collected from tumors. (F) qRT-PCR assay analyzed 3 mRNAs level in miR-653-5p mimics-transfected cells. (G) qRT-PCR and western blot assays examined the mRNA and protein expression of RAI14 after overexpressing miR-653-5p or suppressing AFAP1-AS1. (H) Luciferase reporter assay researched the affinity among AFAP1-AS1, miR-653-5p and RAI14. (I) RIP assay explored the interaction among AFAP1-AS1, miR-653-5p and RAI14. (J) qRT-PCR detected AFAP1-AS1 expression in pcDNA3.1 or pcDNA3.1/RAI14-transfected cells. (K) qRT-PCR measured miR-653-5p level in pcDNA3.1 or pcDNA3.1/RAI14-transfected cells. **P < 0.01. [file 12885_2020_6665_MOESM2_ESM.tif]

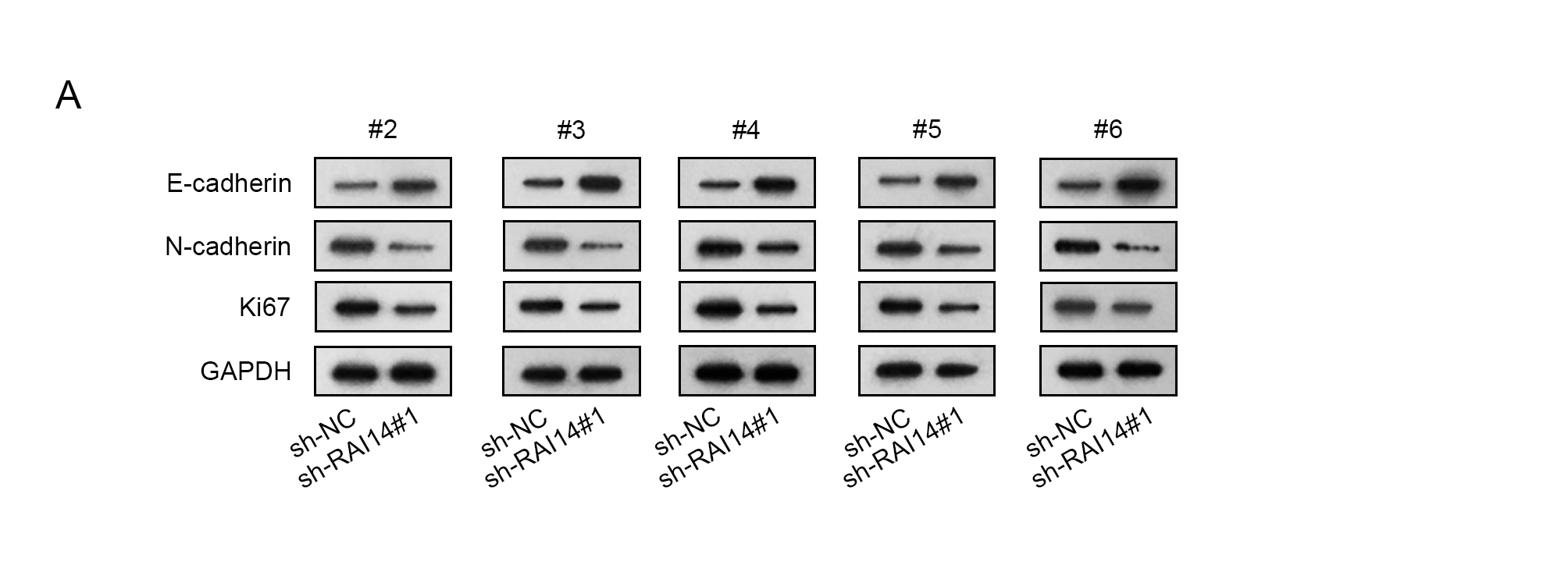

Supplement: Supplementary file 3 — Additional file 3: Supplementary Figure S3. (A) Western blot assay examined Ki67, E-cadherin and N-cadherin protein expressions in sh-NC group or sh-RAI14#1 group in tumors separated from the other 5 tissues of mice. [file 12885_2020_6665_MOESM3_ESM.tif]

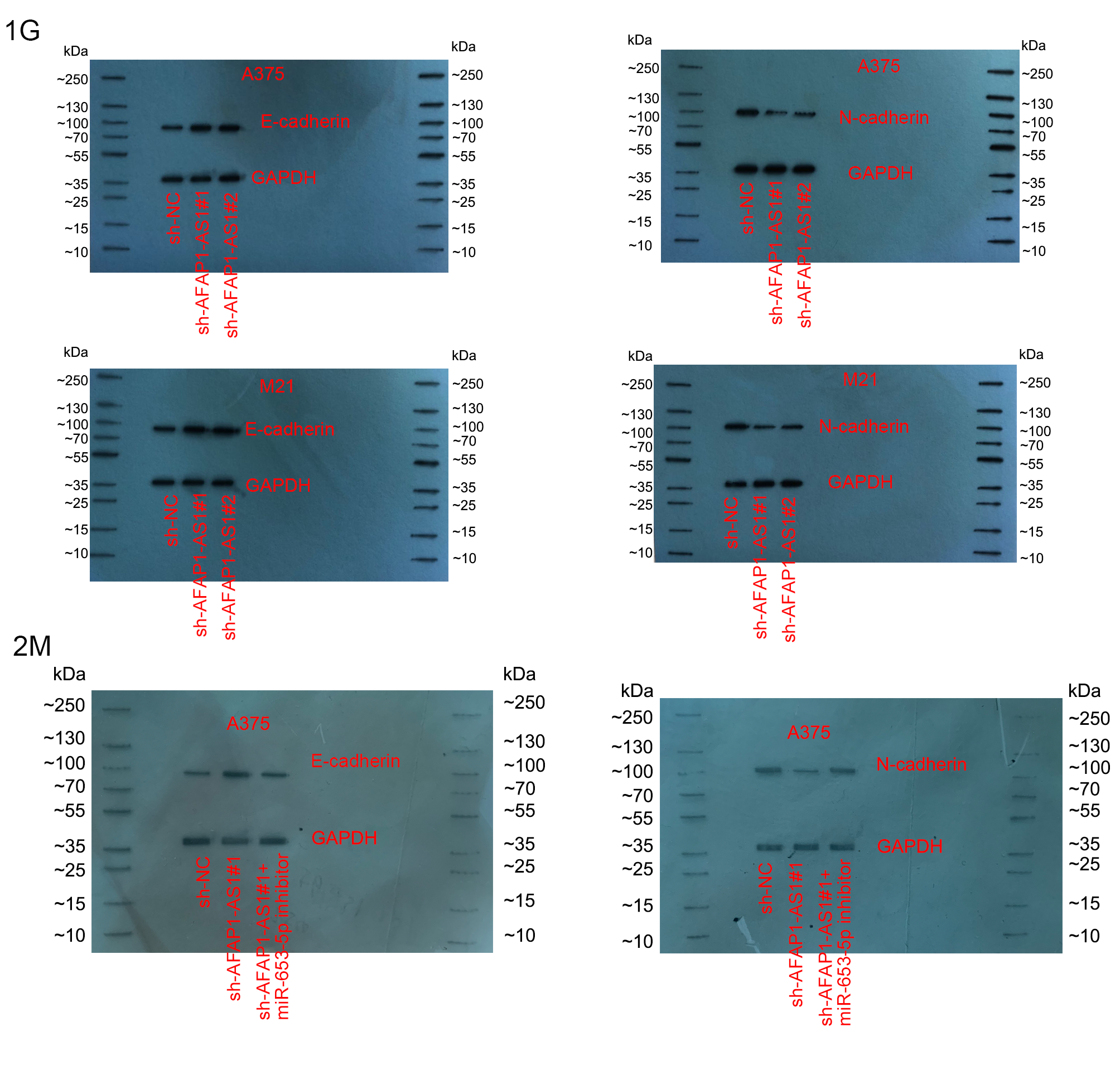

Supplement: Supplementary file 4 — Additional file 4 Supplementary file 1-5 The original western blot data of figure 1G/2M/3C/3D/3 M/4B/4G/S1D/S2E/S2G/S3A were displayed. [file 12885_2020_6665_MOESM4_ESM.zip › supplementary file 1R3.tif]

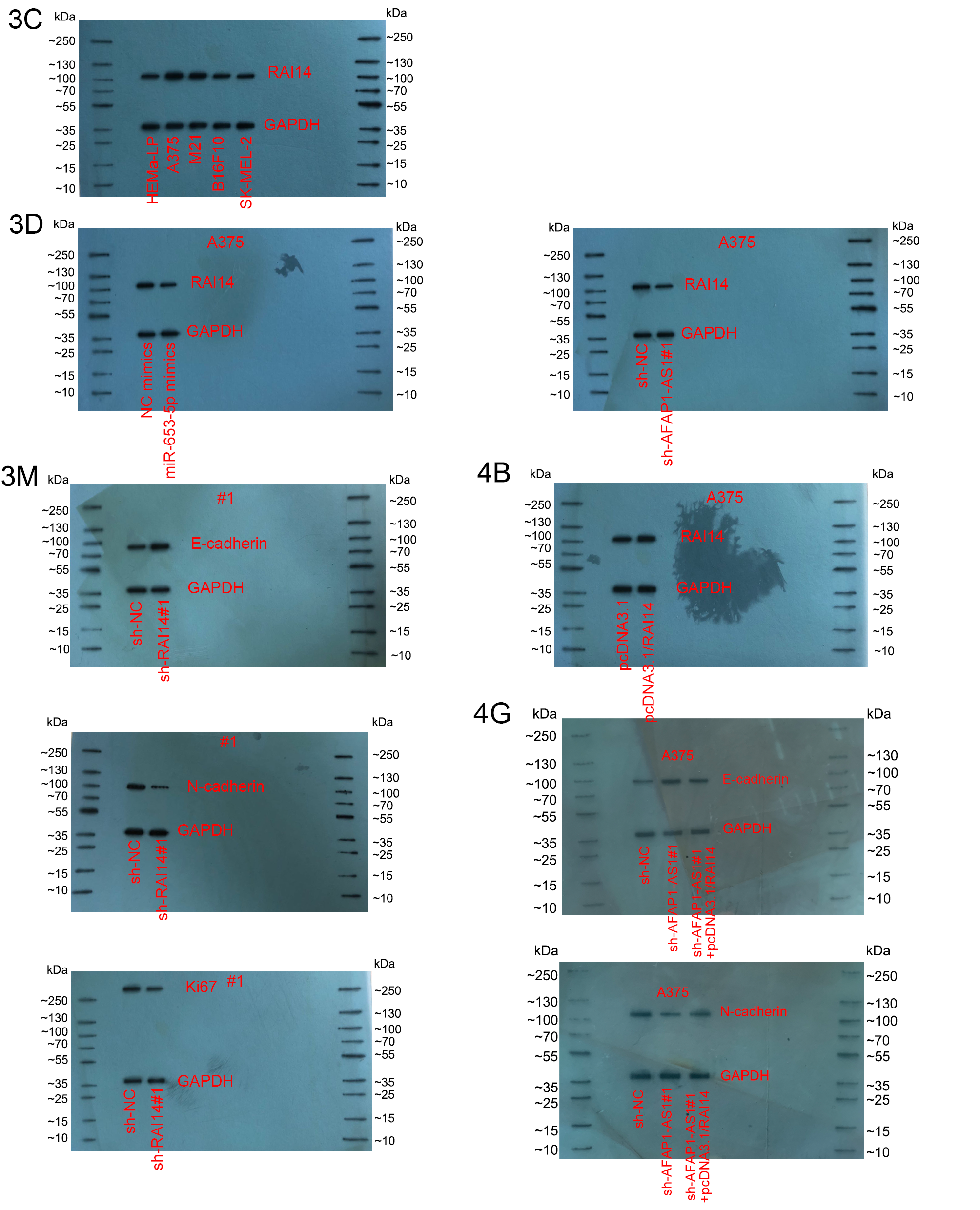

Supplement: Supplementary file 4 — Additional file 4 Supplementary file 1-5 The original western blot data of figure 1G/2M/3C/3D/3 M/4B/4G/S1D/S2E/S2G/S3A were displayed. [file 12885_2020_6665_MOESM4_ESM.zip › supplementary file 2R3.tif]

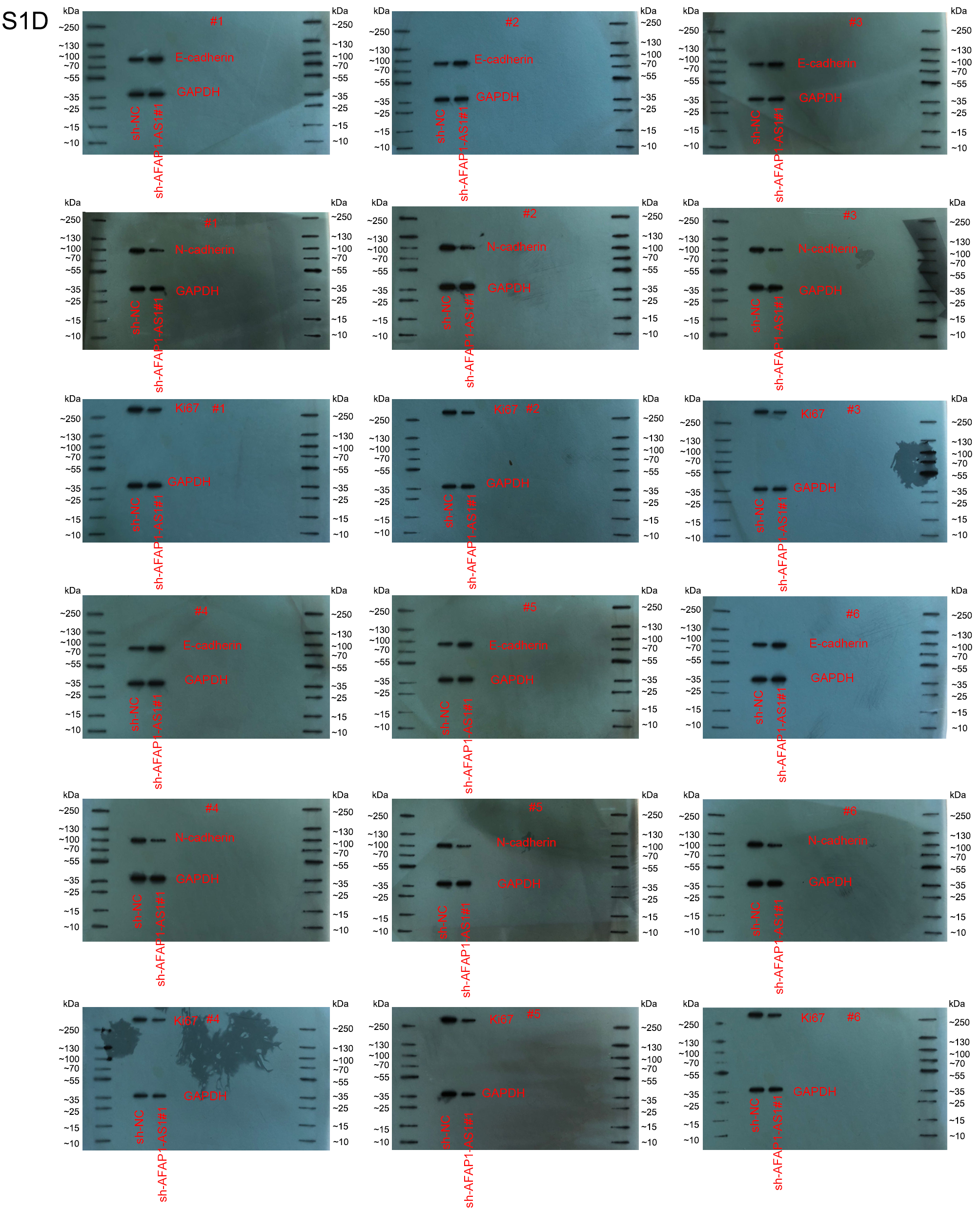

Supplement: Supplementary file 4 — Additional file 4 Supplementary file 1-5 The original western blot data of figure 1G/2M/3C/3D/3 M/4B/4G/S1D/S2E/S2G/S3A were displayed. [file 12885_2020_6665_MOESM4_ESM.zip › supplementary file 3R3.tif]

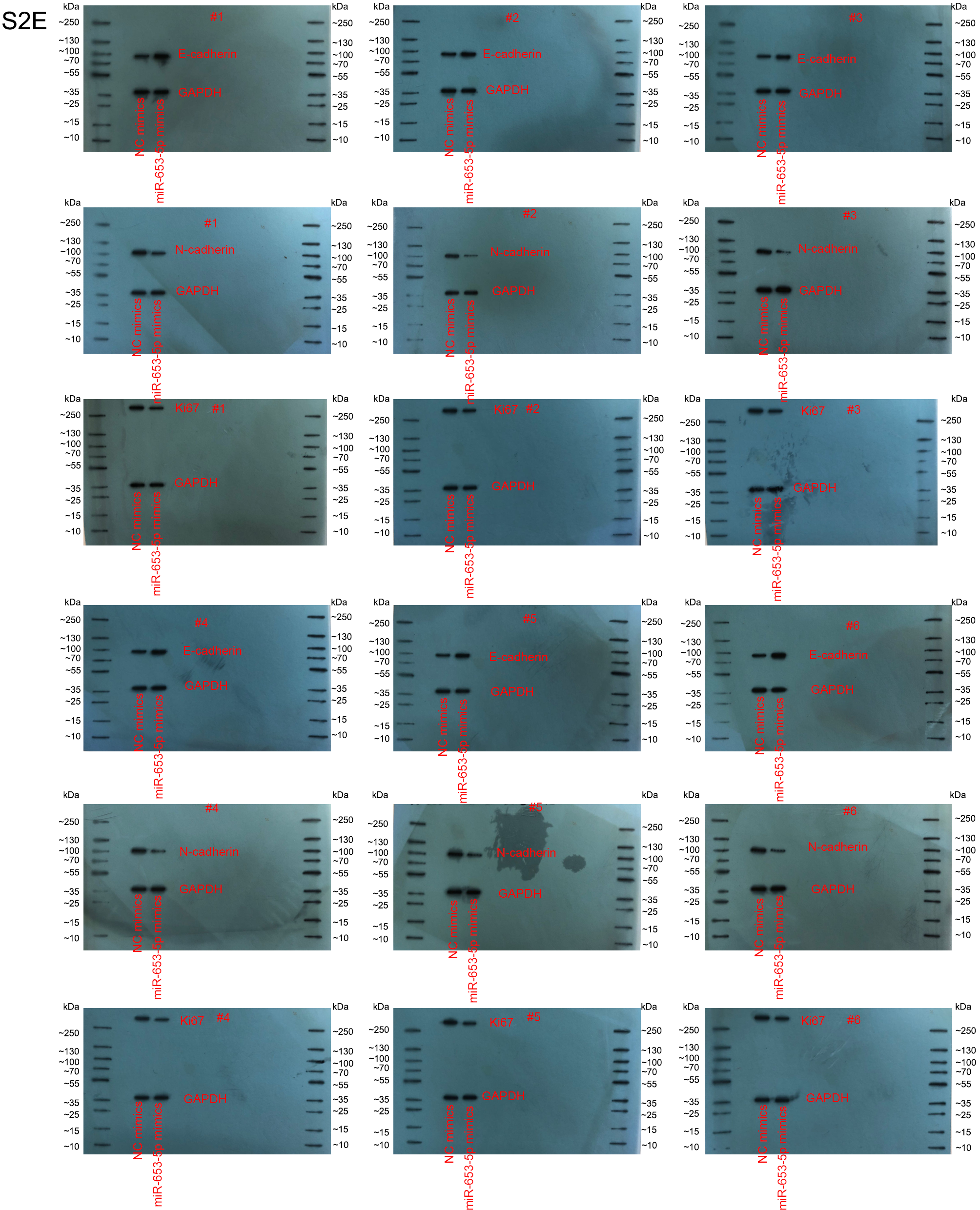

Supplement: Supplementary file 4 — Additional file 4 Supplementary file 1-5 The original western blot data of figure 1G/2M/3C/3D/3 M/4B/4G/S1D/S2E/S2G/S3A were displayed. [file 12885_2020_6665_MOESM4_ESM.zip › supplementary file 4R3.tif]

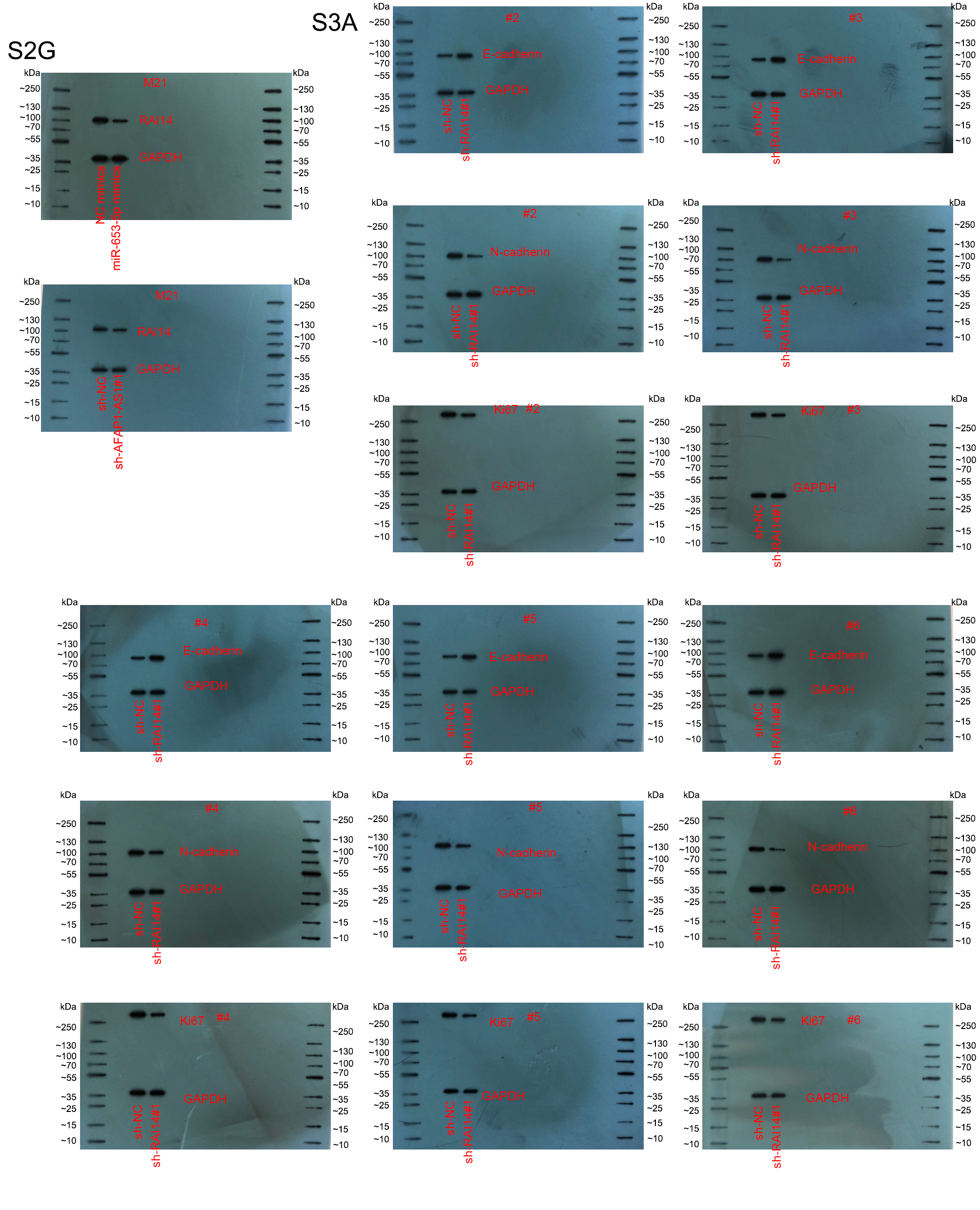

Supplement: Supplementary file 4 — Additional file 4 Supplementary file 1-5 The original western blot data of figure 1G/2M/3C/3D/3 M/4B/4G/S1D/S2E/S2G/S3A were displayed. [file 12885_2020_6665_MOESM4_ESM.zip › supplementary file 5R3.tif]
